# Seeking Structural Solutions

**DOI:** 10.1038/s44172-023-00052-9

**Published:** 2023-01-18

**Keywords:** Engineering

## Abstract

Dr. Zhongyi Zhu shares his passion of solving challenging problems in designing elegant and efficient large-scale spatial structures.

Dr. Zhongyi Zhu is the Chief Engineer of the Beijing Institute of Architectural Design.  He has contributed to more than 20 large-scale construction projects including sports stadiums and large scientific facilities. Here, we talk to Dr. Zhu about how he got started in the field, the challenges of making large scale structures more sustainable, and advice for the next generation of structural engineers.

**Figure:**
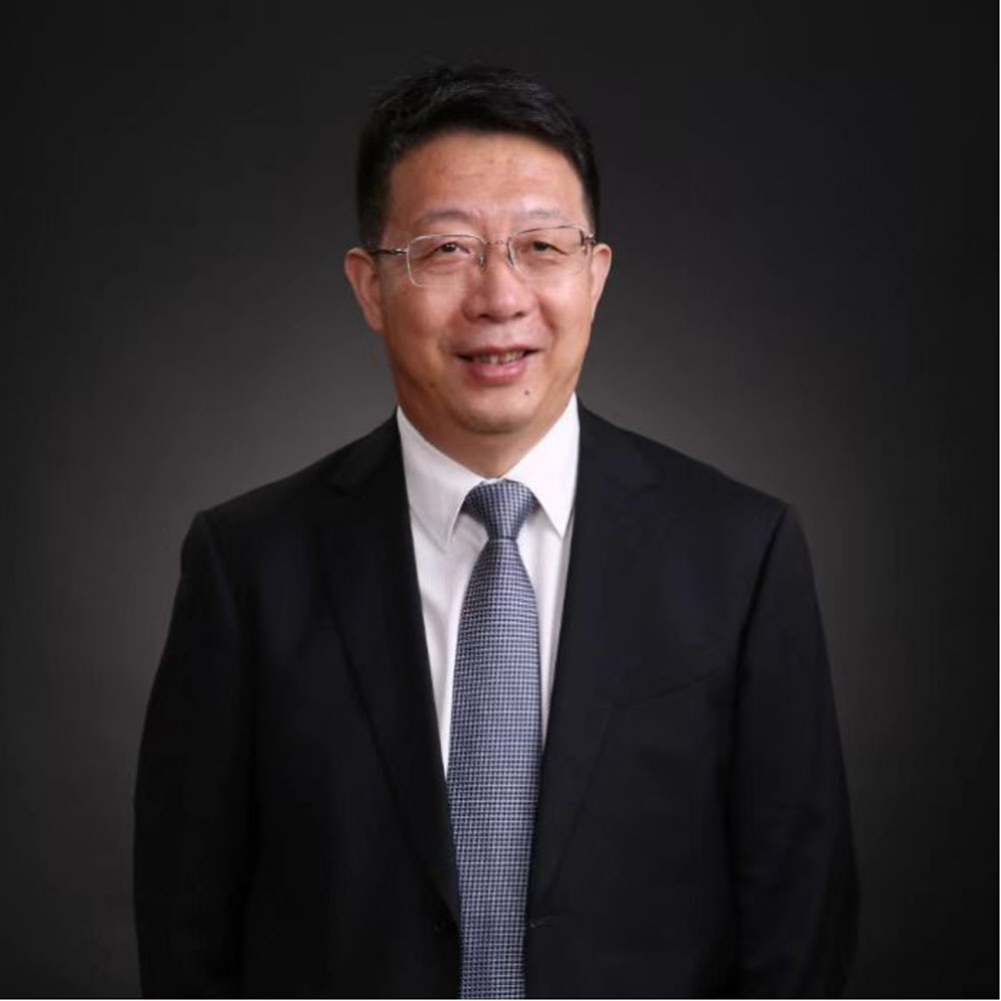
Zhongyi Zhu

1. What originally got you interested in structural engineering?

Around the late 1980s and early 1990s, China embarked on a major program of economic reform and had seen a fast economic growth, which promoted city development and many national construction projects. The plentiful opportunities made civil engineering one of the most popular university engineering majors at the time. And this was the reason I chose civil engineering for my major.

During my undergraduate studies, I took a course named ‘Spatial Structures’, which introduced me to the idea of entirely self-supporting or long span structures, such as space grid and lattice shell structures. I found myself fascinated by these highly efficient and elegant styles. Thus, before graduation, I contacted Dr. Shilin Dong, a famous expert in spatial structures from Zhejiang University, expressing my enthusiasm to join his group as a PhD student. Luckily, I got a positive response. After graduation, I joined Beijing Institute of Architectural Design and started my career as a structural engineer. This is the field I have been focusing on throughout my entire career.

2. Which is your favourite structure from your portfolio and why?

Well, it is a tough question to answer. I put a lot of effort into all my designs and I love them all. One of my favourites that we recently completed is the Five-hundred-metre Aperture Spherical Radio Telescope, now well known as FAST. As the largest single aperture telescope in the world, it is supported by a mega cable structure with a reflecting surface of diameter 500 metres. On one hand, in order to realize astronomical observations, the cable net structure should be able to continuously change shape to move its focus point during the observation. On the other hand, as an astronomy instrument, the design and construction errors of the cable net should be controlled within 1 mm. These features are very different from the traditional cable net structures, and they brought challenges to the design, fabrication and installation. This project expanded the capability of structural engineering and gave me a new vision of what the field could achieve.

**Figure:**
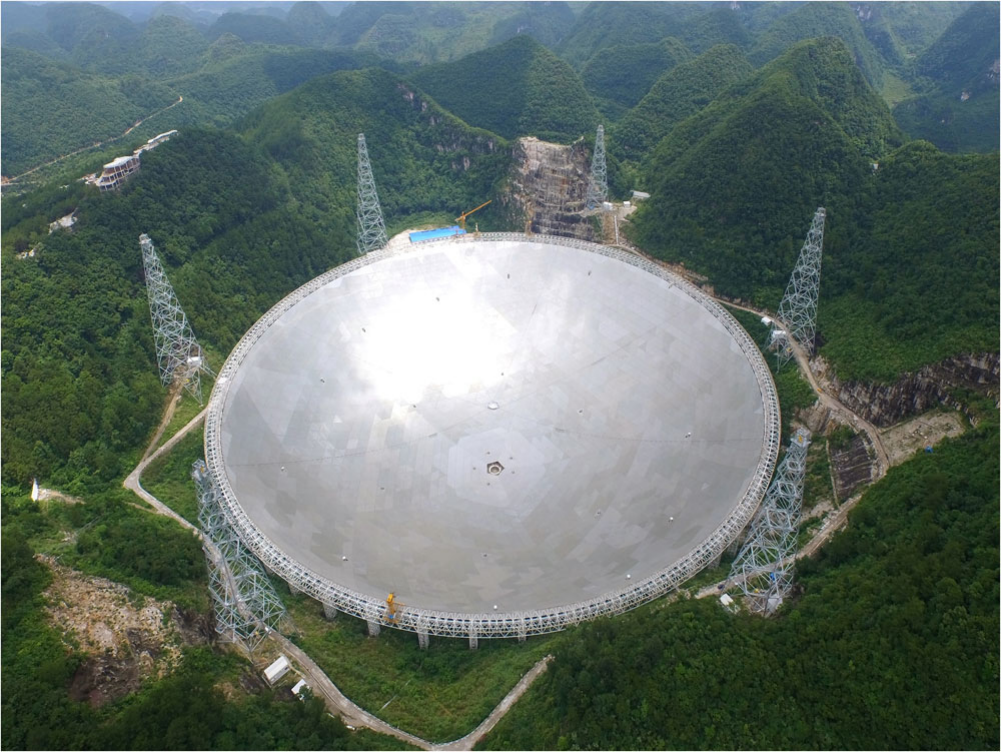
Five-hundred-metre Aperture Spherical Radio Telescope (FAST), China. Reproduced with permission from National Astronomical Observatories of the Chinese Academy of Sciences (NAOC).

3. What do you enjoy most as a structural engineer?

What I enjoy most is the experience of continuously exploring solutions to challenging engineering problems. In the field of megastructure design, often there are no typical examples and established rules or standards to follow. It is a common thing that creative ideas raised by architects break convention and require innovative thinking to find solutions. In most of the projects we design, the geometry of structure should be consistent with the artistic appearance desired by the architect. For instance, cable structures composed of thin and flexible ropes can contribute to the overall architectural effect. This type of structure requires cautious form finding to accurately control its geometry, which is critical but very difficult to achieve using conventional methods. We have successfully controlled the geometry of several very large cable structures with geometric errors less than 1 mm, such as the saddle-shaped cable net roof of China’s National Speed Skating Oval, an iconic venue of Beijing 2022 Winter Olympics, and the Lusail Stadium, the main venue for the Qatar FIFA 2022 World Cup. At the same time, of course, we also need to ensure the efficiency of the cable net structure in the form finding. Our novel designs use flexible cable structures to precisely realize geometries designed by architects, which previously could only be achieved with concrete or steel. Pushing the boundaries of techniques in structural engineering really excites myself and the team. This work is challenging, but I do find my pleasure in it.

4. What steps do you see for structural design to tackle environmental and climate issues?

The primary goal of structural engineering is to design efficient, safe and reliable structures that could satisfy the desired practical functionality and aesthetic creativities of the architectural vision. It is incredibly challenging to introduce sustainability on top as a key design criterion. However I can see several approaches that could be valuable.

The first is the idea of re-usability: structures that are efficient and which can be conveniently assembled and disassembled. For example, I am interested in designing prefabricated long-span structures, which will simplify the operation workload on site and be more convenient for disassembly and further reuse.

Second, we could exploit high performance materials such as carbon fibre, which could reduce material usage and increase durability. Actually we have already designed the roof structure of a stadium in Sanya using carbon-fibre reinforced polymer cable net. We could also explore sustainable structural materials like timber or bamboo. These environmentally friendly materials will reduce carbon emissions.

Another important consideration is how to realize green on-site operation, and maintenance. There is still a lot of work to do to increase efficiency, use less materials and develop simpler construction processes.

5. What advice do you have for the next generation of structural engineers?

Structural engineering is a strong practical subject, requiring qualified and professional engineers which are trained in skills more than can be achieved by reading books. So for younger structural engineers, my advice is to accumulate experience in real construction projects and be an active participant in the project discussion. The best way to gain experience is through working on site.

*This interview was conducted by Mengying Su and Rosamund Daw from the Communications Engineering editorial team*.

